# A Modified Scrotoplasty for Treating Severe Penoscrotal Webbing in Children

**DOI:** 10.3389/fped.2020.00551

**Published:** 2020-09-11

**Authors:** Yuan Li, Xiaoyu Zhu, Dongchuan Feng, Jinchao Gong, Guangyao Sun, Xilun Zhang, Dianhe Hu, Suoyou Sha, Tao Han

**Affiliations:** Department of Urology Surgery, The Affiliated Xuzhou Children's Hospital of Xuzhou Medical University, Xuzhou, China

**Keywords:** child, phimosis, webbed penis, penoscrotal webbing, scrotoplasty

## Abstract

To compare a novel modified W-incision scrotoplasty (MWS) operation method with the conventional V-Y scrotoplasty for treatment of severe penoscrotal webbing (PSW) in children a retrospective study was conducted on 26 children. Circumcision combined with modified scrotoplasty was used to repair the webbed penis and phimosis of children and another 32 patients undergoing V-Y scrotoplasty served as the control group. There was a statistically significant difference of angle improvements of penis and scrotum in a horizontal position (−66 ± 10; −57 ± 6, *P* < 0.001) and the parent satisfaction score (Five Likert Scale) (4.7 ± 0.56; 3.8 ± 0.47, *P* < 0.001) between the two groups. All 26 children who underwent MWS presented with no serious postoperative complications, and there was no significant difference in surgical complications compared to children treated with V-Y scrotoplasty.

## Introduction

Penoscrotal webbing (PSW) is also called congenital penile scrotal fusion and refers to a webbed fusion between the ventral skin of the penis and the median raphe of the scrotum. The webbed penis is a congenital condition in which a web or fold of skin between the penis and the scrotum obscures the penoscrotal angle in an otherwise normal-sized penile shaft (1–3). It is easily confused with concealed penis, micropenis and trapped penis. PSW can also occur as a concomitant symptom of some diseases. As a concomitant symptom, in many cases PSW is often not diagnosed.

In 2010, El-Koutby and Mohamed Amin (4) proposed three classifications of PSW, namely simple, compound and secondary webbed penis, respectively. Compound webbed penis mostly presents with the penile body partially or completely enclosed by scrotal skin. Secondary webbed penis is mostly caused by removing too much skin at circumcision resulting in too little skin preservation on the ventral side of the penis. Simple webbed penis is characterized only by webbed fusion between the ventral skin of the penis and the median raphe of the scrotum. According to the range covering the penis body, simple PSW can be further divided into: Grade 1: The web extends to the proximal 1/3 of the shaft of the penis; Grade 2: The web extends to the mid 1/3 of the penis; Grade 3: The web extends to the distal 1/3 of the penis.

At the end of the 19th century, Heineke-Mikulicz reported the use of transverse incision and longitudinal sutures to treat partial fusion of the penis and scrotum. After various improvements, double-V scrotoplasty (DVS), V-Y scrotoplasty, wedge excision scrotoplasty and Z scrotoplasty were developed (5–10) but still the merits and demerits of various surgical methods are disputed (10). In the 2018 Guidelines on pediatric day surgery of the Italian Societies of Pediatric Surgery (SICP) and Pediatric Anesthesiology (SARNePI), there is new guidance for a PSW operation. An abnormal peno-scrotal junction, resulting in a ventral web, is not only an esthetic problem but it can involve a functional complication during erection. The common V-Y or multiple Z plasty are easily performed as day surgery procedures (11).

In recent years, the above-mentioned surgical methods have been used in the treatment of congenital penile-scrotal fusion in China, but a systematic comparison of the two surgical methods has not been reported.

In the present study, 26 children each with a severe webbed penis complicated with phimosis were treated with MWS plus circumcision, and their efficacy and adverse reactions (complications during follow-up) were compared with those of children treated with V-Y scrotoplasty. The aim was to confirm the effectiveness of the new MWS technique in the treatment of severe congenital PSW with phimosis in children.

## Patients and Methods

### Clinical Data

In this paper, we have summarized 26 cases of children with a simple type of PSW from July 2012 to April 2018. All the children underwent MWS combined with circumcision. The average age at surgery was 5 years and 5 months (year range: 1–14), and the patients were followed-up for at least 6 months. Inclusion criteria were according to the standard diagnosis in the 11th edition of CAMPBELL-WALSH UROLOGY in 2016 diagnosed congenital penile and scrotal fusion requiring surgical treatment with no serious deformities of other systems and no other penis-related diseases. Exclusion criteria were other penile deformities found during the operation and children with incomplete postoperative data. We compared outcomes with 32 PSW children previously treated with V-Y scrotoplasty and the study was double-blinded during the follow-up of postoperative complications and the survey of children's parental satisfaction.

### Grading

According to the range covering the penis body, simple PSW has been divided into a moderate group comprising Grade 1 in which the web extends to the proximal 1/3 of the shaft of the penis and Grade 2 in which the web extends to the mid 1/3 of the penis as well as a severe group in which the web extends to the distal 1/3 of the penis (Grade 3) (4).

### Parents Satisfaction Score

The survey of parents' satisfaction refers to a Five Likert Scale. The survey consists of statements regarding penile size, morphology, voiding status and hygiene with the 5 grades for each item: 5, very satisfied; 4, satisfied; 3, average; 2, dissatisfied; 1, very dissatisfied (12).

### V-Y Scrotoplasty

A V-shaped incision was made on the ventral side of the penis. The ventral webbed skin was cut longitudinally along the midline as the long axis, with the new penis scrotal angle as the apex. The fusion of penis and scrotum on the ventral side of the penis body initially moves down to a new angle of penis and scrotum. Pruning downward penile scrotal fusion incisions were made on both sides. Any wrinkled skin was removed and intermittent sutures were applied. Detailed sketches of the operation are available in the study of Bonitz and Hanna (10).

### Statistical Analyses

SPSS version 17.0 (IBM, Armonk, NY, US) was used for all statistical analysis. Continuous data are described as means ± standard deviation, and any differences compared using two-sample *t*-tests. Categorical data are given as counts and proportions and were analyzed using a chi-squared test. *P* < 0.05 were considered to be statistically significant.

## MWS Method

### Surgical Methods

**Figure 1:**
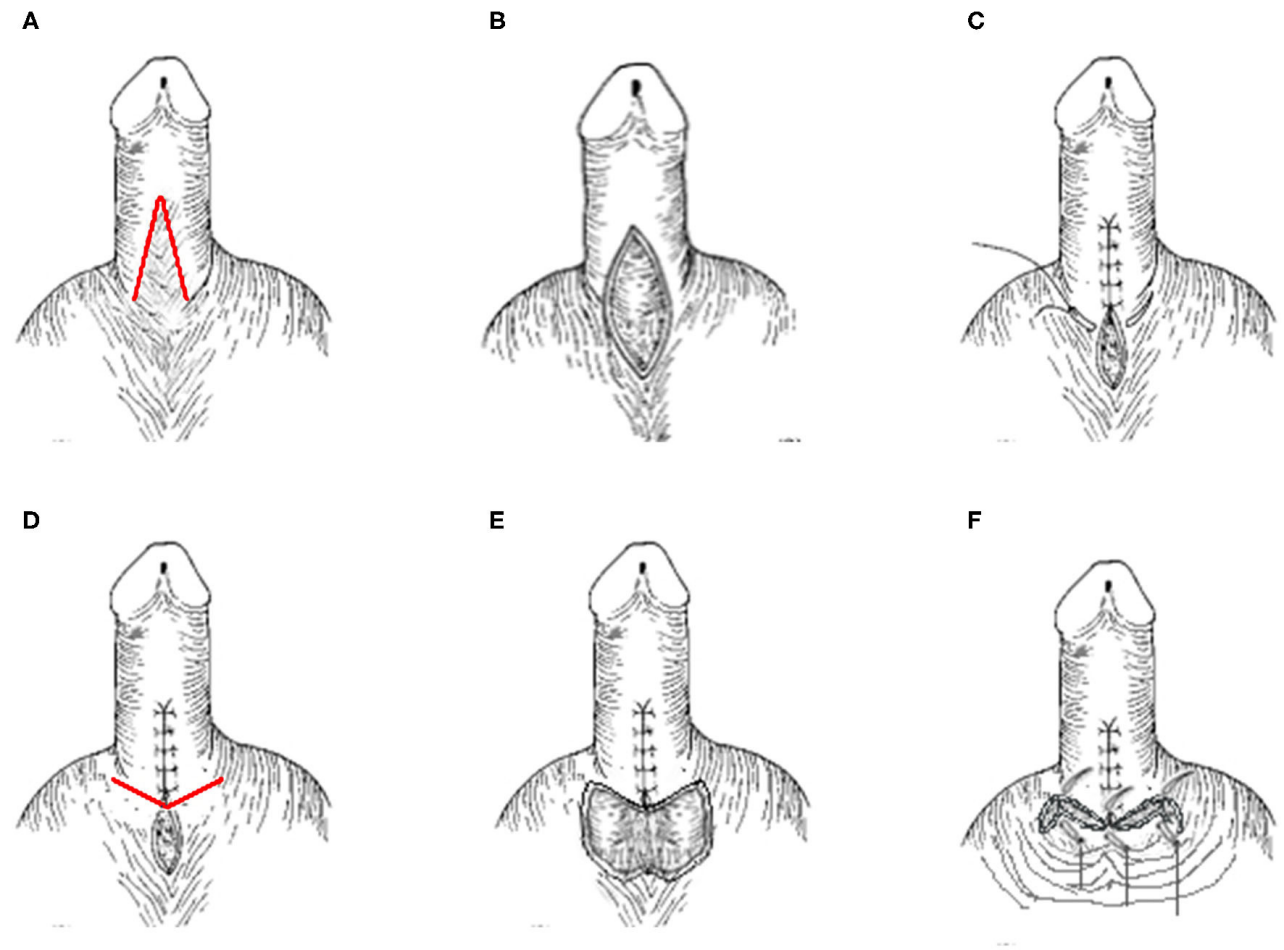
MWS approach for PSW treatment. **(A)** An inverted V-shaped incision was made along the penis-scrotum fusion (the red line). **(B)** The fused part of the penis and scrotum was isolated and **(C)** the penile scrotal angle was reconstructed. **(D)** A V-shaped incision along the newly created penis scrotum angle was created (the red line), **(E)** the skin at the junction of the penis and scrotum was cut, and **(F)** after which the remaining penis-scrotum fusion section was sutured to cover the wound.

First, an inverted V-shaped incision was made along the penis-scrotum fusion (Figure 1A, the red line). Then, the fused part of the penis and scrotum was isolated (Figure 1B) and the penile scrotal angle was reconstructed (Figure 1C). Then a V-shaped incision along the newly created penis scrotum angle was created (Figure 1D, the red line) and the skin at the junction of the penis and scrotum was cut (Figure 1E), after which the remaining penis-scrotum fusion section was sutured to cover the wound (Figure 1F).

Finally, the dorsal center of the foreskin was cut to 1 cm from the coronary sulcus. After the foreskin was loosened and an inner Shang ring positioned, the foreskin was everted over the inner ring. Then, the outer ring was placed over the inner ring thereby sandwiching the foreskin. Finally, the foreskin was removed and the incision was pressurized and bandaged (Shang Ring, Disposable circumcision anastomat and push-off assembly, WuHu Snnda Medical Treatment Appliance Technology Co. Ltd. Wuhu, China).

## Results

Table 1 shows the baseline characteristics of the 2 groups. The patients in the MWS group included significantly more Grade 3 PSW cases (*P* < 0.001, Table 1).

**Table 1:** Baseline characteristics, relevant pathologies, and the type of children with PSW.

	**MWS (*n* = 26)**	**V-Y scrotoplasty (*n* = 32)**	***P*-value**
Age of children (months)	69.5 ± 13.6	70.9 ± 12.7	0.687
Grading of webbed penis *N*, %			
Severe (Grade 3) (*N*, %)	19 (73.1)	6 (18.8)	<0.001
Moderate (Grade 1 and 2) (*N*, %)	7 (26.9)	26 (81.3)	

The angle of the penis and scrotum in the horizontal position before surgery did not differ significantly between the two groups. However, the pre-post surgery differences of the angle of the penis and scrotum in the horizontal position differed significantly (*P* < 0.001). Also, the parent satisfaction score was significantly better in the MWS compared to the V-Y scrotoplasty group (*P* < 0.001) (Table 2).

**Table 2:** Operation process, curative effect, and complications at 6 months follow-up.

	**MWS (*n* = 26)**	**V-Y scrotoplasty (*n* = 32)**	***P*-value**
Surgery time (min)	28 ± 3.2	26 ± 2.4	0.009
Success rate %	100	100	
**Pre-surgery**			
The angle of penis and scrotum in horizontal position (cm)	134°± 20	131°± 18	0.551
**After Surgery**			
The angle of penis and scrotum in the horizontal position	68°± 10	74°± 12	0.046
Δ Angle of penis and scrotum in the horizontal position	−66 ± 10	−57 ± 6	<0.001
Parent satisfaction score (Five Likert Scale)	4.7 ± 0.56	3.8 ± 0.47	<0.001
**Surgical Complications**			
Hemorrhage (*N*, %)	2 (7.7)	1 (3.1)	0.582
Infection (*N*, %)	0 (0)	1 (3.1)	1.000
Skin splitting caused by tension in the incision (*N*, %)	0 (0)	4 (12.5)	0.681
Scar hyperplasia (*N*, %)	2 (7.7)	2 (6.3)	1.000
Penile edema (*N*, %)	0 (0)	4 (12.5)	0.681
Re-operation (*N*, %)	0 (0)	0 (0)	
Total (*N*, %)	4 (15.4)	12 (37.5)	0.080

*Δ: Change between post-treatment and pre-treatment*.

No obvious swelling of the prepuce was detected and the incisions in 2 cases slightly bled in the MWS group, and two children had mild scar hyperplasia at the angle of the penis and scrotum. However, surgical complications were not significantly different between the 2 groups (Table 2).

**Figure 2:**
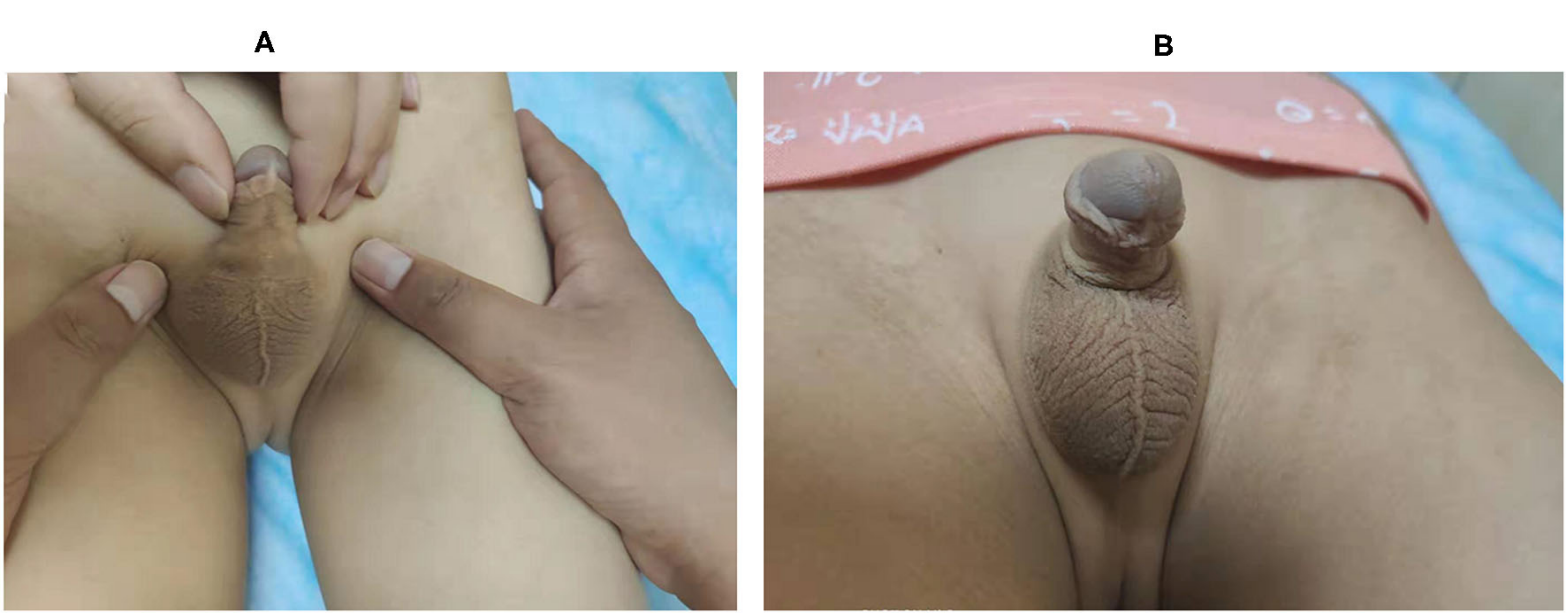
Post surgery outcomes of MWS treatments. **(A)** Incision healing in 1 year after operation. **(B)** Penile scrotal angle morphology 1 year after surgery.

One year after surgery, the incisions were properly healed and the penile scrotal angel was improved in all MWS patients (Figure 2).

## Discussion

MWS was easy to carry out because scrotoplasty with combined circumsicion was simpler and easier to operate than scrotoplasty with degloving prepuce as used in the past. PWS cases included in our study were mostly found when the patients visited their doctors with phimosis or penis appearance dissatisfaction; they were all PWS cases combined with phimosis. It is noteworthy that the choice of surgical indications for such children and patients is highly controversial (13, 14). Previous conclusions indicated that the disease had no clinical symptoms and did not require surgical treatment, at least in childhood. However, recent studies have found that many PSW children and their parents suffer from psychological or social pressure because of the unsatisfactory appearance of the penis during their growth. Some severe cases have adult problems such as sexual intercourse disorder, difficulty in using a condom and/or profound effects on sexual self-esteem (15–18). In the clinic, it is necessary to treat this kind of simple webbed penis with phimosis, since a comparative study conducted by Herndon et al. (19) confirmed that the quality of life of children with webbed penis treated surgically in childhood was significantly improved compared with those treated in their youth (19). In the past, the most commonly used surgical method for simple PWS cases combined with phimosis was circumcision plus double V or V-Y scrotoplasty, which were easy to operate and significantly improved the postoperative appearance.

In a previous study, however, it was noted that DVS, which is a modification of the V–Y technique designed to improve cosmetic results (9) may cause increased skin separation, due to the increased tension at the penoscrotal junction. V-Y scrotoplasty should be undertaken only for mildest PSW cases otherwise Z scrotoplasty was mainly used for more severe cases (10). We have made some improvements on the basis of V-Y scrotoplasty. After reconstruction of the new penoscrotal angle, the scrotal skin was no longer removed. Instead, the new penoscrotal angle of the penis was taken as the vertex and a V-shaped incision was made along the junction between the upper edge of the scrotum and the skin to both sides, thereby reducing the tension at the penoscrotal angle. The origin of the ventral penoscrotal fusion moves down to the new penoscrotal angle, retaining the scrotal skin to cover the incision. A W incision effectively reduces the tension at the angle of the newly created penis and scrotum, reducing the possibility of incision dehiscence and scar hyperplasia. At the same time, retaining the scrotal skin not only avoids a poor scrotal shape after resection, but also hides the incision at the junction of the penis and scrotum, and has high appearance satisfaction (Supplementary Figure 1). In terms of surgical complications, there was no significant difference between the two groups, but in terms of a trend the surgical complications in the MWS group were less than those in the V-Y scrotoplasty group. These observations may be related to the small sample size, and in future studies the size of the cohort of patients will be increased. In terms of surgery time, MWS takes significantly longer than V-Y scrotoplasty to implement, but it does produce a more acceptable outcome. In the MWS group, angel improvement of the penis and scrotum in a horizontal position were significantly better than in V-Y scrotoplasty patients, which might be explained by the fact that the MWS group consisted of significantly more Grade 3 PWS cases.

The limitations of the present study was the relatively small number of cases, and that due to the poor compliance of children, the measurement results were prone to errors.

## Conclusions

We used circumcision combined with MWS to repair mostly severe PSW in 26 children resulting in a good postoperative appearance, little trauma, high parent satisfaction and easy clinical implementation. The MWS is an alternative approach for V-Y scrotoplasty, which might be used for severe PSW cases.

## Data Availability Statement

The datasets generated for this study are available on request to the corresponding author.

## Ethics Statement

The studies involving human participants were reviewed and approved by the Ethical Committee of Xuzhou Children's Hospital affiliated to Xuzhou Medical University. Written informed consent to participate in this study was provided by the participants' legal guardian/next of kin.

## Author Contributions

YL, XZhu, and TH were responsible for the conception and design of the study. YL, XZhu, DF, and JG were responsible for acquisition and analysis of data. Furthermore, GS, XZha, DH, and SS were in charge of statistical analysis. YL drafted the manuscript. YL and TH revised and commented the draft. All the authors approved the final version of the manuscript.

## Conflict of Interest

The authors declare that the research was conducted in the absence of any commercial or financial relationships that could be construed as a potential conflict of interest.

## Supplementary Material

Supplementary Figure 1 Lateral images of **(A)** preoperative, **(B)** intraoperative, **(C)** postoperative, and **(D)** 1-year follow-up of a child with webbed penis.
