# Supplementary figures and images for: A Modified Scrotoplasty for Treating Severe Penoscrotal Webbing in Children
Source: Front Pediatr. 2020 Sep 11;8:551. doi: 10.3389/fped.2020.00551 (PMC7533638; doi:10.3389/fped.2020.00551)

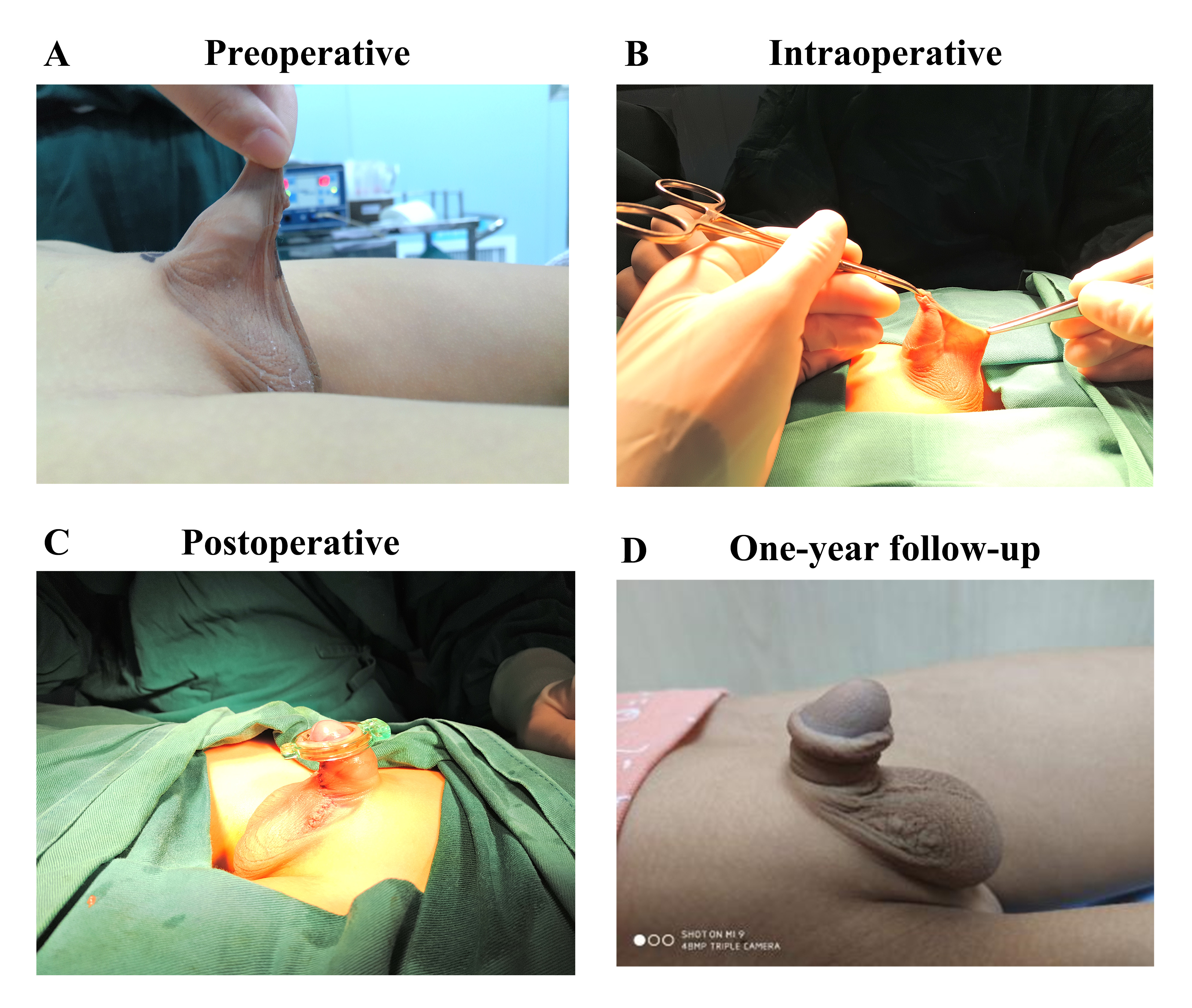

Supplement: Supplementary Figure 1 — Lateral images of (A) preoperative, (B) intraoperative, (C) postoperative, and (D) 1-year follow-up of a child with webbed penis. [file Image_1.JPEG]
